# Genome-wide association studies builds a predictive model and reveals novel resistance features for macrolide-resistant *Bordetella pertussis*

**DOI:** 10.1093/jac/dkag131

**Published:** 2026-04-16

**Authors:** Jialiang Chen, Guowei Liang

**Affiliations:** Department of Clinical Laboratory, Aerospace Center Hospital, Beijing 100049, China; Department of Clinical Laboratory, Aerospace Center Hospital, Beijing 100049, China

## Abstract

**Objectives:**

The macrolide resistance in *Bordetella pertussis* cannot be fully explained by 23S rRNA mutations, underscoring the need for comprehensive methods to detect resistant isolates and clarify mechanisms.

**Methods:**

Whole-genome sequencing data from 556 isolates with macrolide resistance information, including 398 resistant and 158 sensitive strains, were retrieved from the National Center for Biotechnology Information (NCBI). A k-mer-based genome-wide k-mer-based association studies using Pyseer identified 1322 resistance-associated k-mers. Refinement with Scoary2, least absolute shrinkage and selection operator (LASSO) and variable selection using random forests (VSURF) yielded six key k-mers, enabling the construction of a simplified model for predicting resistance.

**Results:**

A total of 1322 different k-mers were involved in resistance. In the further simplified model, only six k-mers were included, in which a DHCW motif cupin fold protein (odds ratio [OR]: 25.84, 95% confidence interval [CI]: 15.58–44.03) and an *IS481* insertion sequence located near *infA* (OR: 28.96, 95% CI: 7.64–243.86) showed strong associations with resistance. Despite the reduced feature set, the simplified model achieved classification performance comparable to the initial model, with similar sensitivity (97.7% versus 98.7%), specificity (92.1% versus 99.5%), and accuracy (93.4% versus 99.3%). Notably, it maintained a robust area under the receiver operating characteristic curve (area under the curve = 0.98), indicating strong predictive capability.

**Conclusions:**

This study developed a simplified k-mer-based model for accurately identifying macrolide-resistant *B. pertussis* isolates and uncovered novel resistance features.

## Introduction

Pertussis, a highly contagious respiratory disease caused by *Bordetella pertussis*, is characterized by prolonged severe coughing that can lead to life-threatening complications in infants.^1^ In the USA, the incidence of pertussis fell from 150–200 to 0.5–1 case per 10 000 population after the diphtheria–tetanus–pertussis (DTP) vaccine’s inclusion in the Expanded Programme on Immunization in 1974, mirroring the global decline.^2^

In China, pertussis dropped from 1000–2000 to <2 per 10 000 by 2006–2013 after whole-cell pertussis vaccine (wP) introduction, with <3000 cases annually.^3^ Despite >99% DTP coverage, cases resurged by 2024 due to pathogen changes and waning immunity.^4^ The Chinese CDC reported 32 380 cases in the first 2 months of 2024—a 23-fold increase over the same period in 2023,^5^ likely an underestimate due to incomplete surveillance. Similarly, US cases rose from 7063 (21.1/10 000) in 2023 to 35 435 (106.3/10 000) in 2024, indicating a global resurgence.^6^

Macrolide antibiotics—including azithromycin, erythromycin, roxithromycin and clarithromycin—are the first-line treatment for pertussis. However, resistance in China is severe, with rates 70%–100%.^7,8^ Li *et al*. reported 91.9% resistance in 2013–2014 isolates and 87.5% erythromycin resistance from 2014 to 2016 (292/335 strains).^9^ Excessive antibiotic use drives resistance, highlighting the need for rapid resistance profiling before treatment. Due to culturing challenges, quick detection methods for *B. pertussis* are urgent.

Resistance mainly involves an A2047G mutation in 23S rRNA domain V, reducing drug binding.^10^ While common, some resistant strains lack this mutation, suggesting other mechanisms, though hypotheses like erm methylase involvement remain unproven.^11,12^ In this study, Genome-wide k-mer-based association studies (GWAS) were used to identify resistance-associated k-mers, which were then refined using Scoary2, least absolute shrinkage and selection operator (LASSO) and variable selection using random forests (VSURF).^13,14^ A simplified k-mer set was used to construct a predictive model for macrolide resistance, revealing novel molecular signatures and offering new tools for detecting resistant strains and understanding resistance mechanisms in *B. pertussis*.

## Materials and methods

### Sequencing of B. pertussis and phenotypic data

Sequence data of 556 *B. pertussis* isolates were obtained from the National Center for Biotechnology Information (NCBI) database (https://www.ncbi.nlm.nih.gov/), and studies containing phenotypic data on isolates’ resistance to macrolide antibiotics were investigated by searching PubMed.^15–18^ In addition to grouping patients based on macrolide-resistance or macrolide-sensitivity, they were stratified by gender and age of patients and isolation year, and region of these isolates. The reference genome used in this study is GCF_004008975.1.

### Assembly, Phylogenetic analyses and typing analyses

For bacterial next-generation sequencing data, the SRA data were first downloaded and converted into paired-end FASTQ files using prefetch and fasterq-dump from the SRA Toolkit (v3.2.0, https://github.com/ncbi/sra-tools). The raw data quality was then assessed with FastQC (v0.12.1, https://github.com/s-andrews/FastQC). Quality control was performed using Trimmomatic (v0.33, https://github.com/timflutre/trimmomatic) to retain high-quality reads. After trimming, FastQC was rerun to confirm the improvement in data quality. Subsequently, genomes were assembled using SPAdes (v4.1.0, https://github.com/ablab/spades). The quality of the assembled genomes was evaluated using QUAST (v5.2.0) and CheckM (v1.2.2). Only high-quality drafts with completeness >90% and contamination <5% were used for downstream analysis. Genomes were annotated using Bakta (v1.9.3, https://github.com/oschwengers/bakta). Pan-genome analysis was then performed using Roary (v3.13.0, https://github.com/sanger-pathogens/Roary), and neighbour-joining (N-J) trees were constructed using FastTree (v2.1.10, https://github.com/morgannprice/fasttree) based on the alignment of core genes. Genotyping of key vaccine antigen genes (*ptxA*, *ptxP*, *fim2*, *fim3* and *prn*) was performed using BLAST (v1.4.0, https://github.com/ncbi/elastic-blast) analysis. Reference sequences were downloaded from the NCBI database (Table S1, available as Supplementary data at *JAC* Online).

### k-mers GWAS

9- to 100-bp k-mers length were scanned from all genomic assemblies by fsm-lite (v1.0, http://github.com/nvalimak/fsm-lite). Differential k-mers potentially associated with macrolide-resistance were initially identified using Pyseer (v1.3.11, https://github.com/mgalardini/pyseer). To ensure specificity, we applied a stringent filter: we only considered k-mers valid if they occurred within a syntenically conserved ‘triple-gene’ block (Upstream–Target–Downstream). To visualize the global genomic distribution significant hits (*P* < 8.34E-09, Bonferroni-corrected for ∼6 million variants) were mapped to the reference genome (GCF_004008975.1) using annotate_hits_pyseer and plotted using ggplot2.

To ensure the robustness of our results and minimize false positives inherent in single-algorithm approaches, we implemented a consensus feature selection strategy using three complementary methods: Scoary2 (v0.0.15, https://github.com/MrTomRod/scoary-2) for statistical association, LASSO (https://github.com/SoftwareObservatorium/lasso) for regularized linear regression, and VSURF (v1.1.0, https://github.com/robingenuer/VSURF) for non-linear importance ranking. In the LASSO analysis, 10-fold cross-validation was used to determine the optimal penalty; features were retained based on lambda.min (the lambda giving the minimum mean cross-validated error) and lambda.1se (the most parsimonious model within one standard error of the minimum) to balance sensitivity and model simplicity. In VSURF, k-mers were prioritized using the MeanDecreaseGini (MDG) metric. The final importance scores were derived from a resubstitution Random Forest model trained on the consensus features with specific parameters: ntree = 500, mtry = 2, nodesize = 1, seed = 123 and sampling with replacement enabled. MDG measures the average reduction in node impurity (Gini index) contributed by a k-mer across all trees, with higher values indicating greater importance for classifying sensitivity versus resistance. K-mers were then sorted in descending order of their MDG scores to generate the importance ranking. This intrinsic metric was chosen for its direct interpretation of feature contribution and computational efficiency.

**Figure 1.**
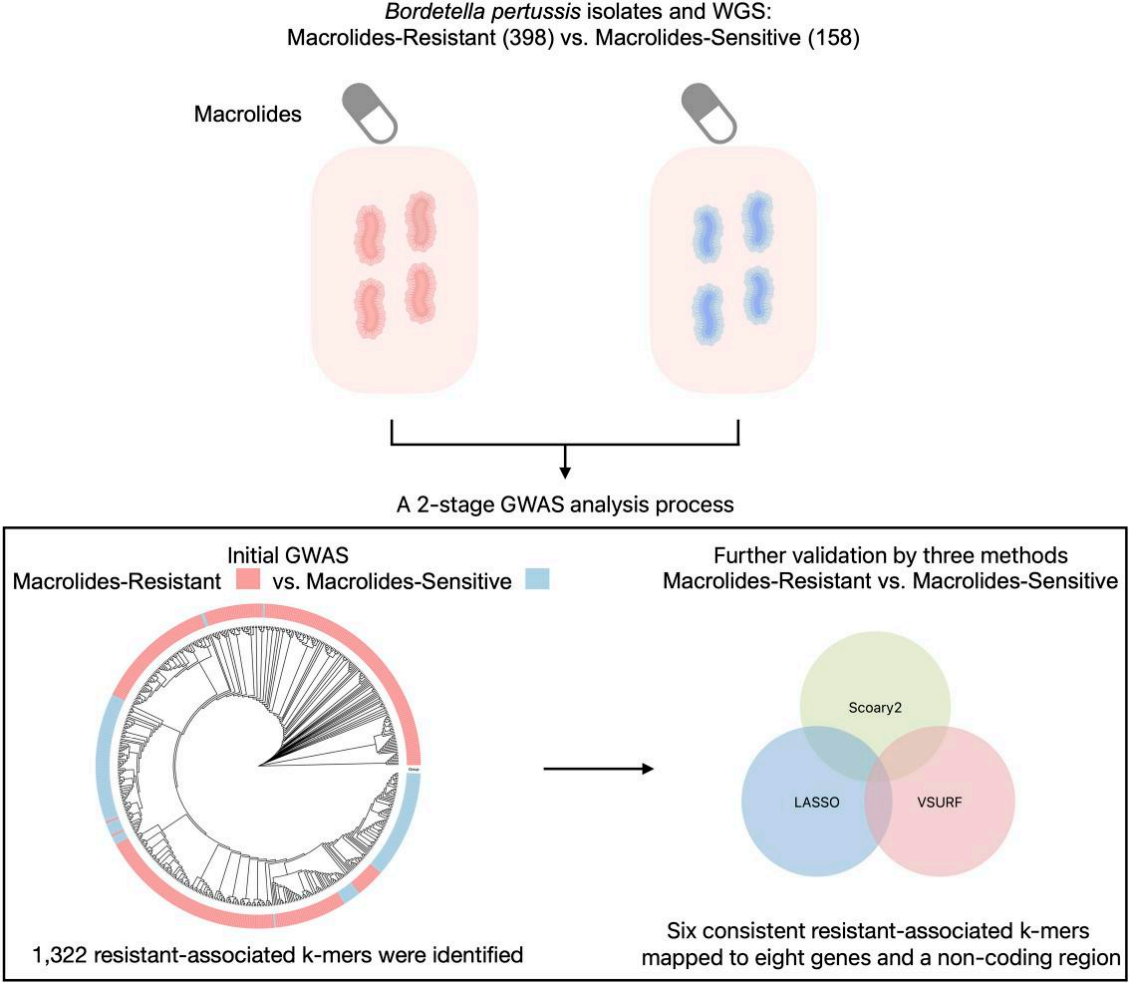
The schematic figure of the GWAS analysis and simple model construction process in this study.

Separately, for high-resolution functional characterization and to broadly survey the genetic landscape, we annotated the top 100 k-mers ranked by their MDG scores derived from the VSURF analysis of all 1322 k-mers. These top-ranked k-mers, along with the consensus biomarkers, were annotated using annotate_hits_pyseer to determine genomic context (coding versus intergenic) and assigned functional categories based on UniProt predictors. A flowchart showing the GWAS analysis and model construction process. Starting from k-mer detection, followed by association analysis, then variable selection using Scoary2, LASSO and VSURF, and finally model building for macrolide resistance prediction (Figure 1).

### Statistical analysis and data visualizations

All statistical analyses and data visualizations were performed using R software (v4.4.3). Graphical plots were generated using the packages ggplot2 for general visualization, ComplexHeatmap for heatmap construction, and UpSetR for UpSet plots. Between-group comparison of categorical variables was performed using the chi-square test (χ2 test) and Fisher’s exact test. Unadjusted odds ratios (ORs) and 95% confidence intervals (CIs) were estimated using the conditional maximum likelihood method, and associations between variables were tested using Fisher's exact test via the fisher.test function in R software. A *P*-value of <0.05 was considered statistically significant.

## Results

### Characteristics of B. pertussis isolates

A total of 556 *B. pertussis* genomes were collected, including 398 macrolide-resistant and 158 macrolide-sensitive strains. The phenotypic data and patient demographics for these isolates are detailed in Table S2. The isolation years of the isolates ranged from before 1980 to 2022, covering 21 provinces across China. The age of patients ranged from less than 1 month to 9 years. The macrolide-resistant group exhibited a male-to-female ratio of 1.32:1 (194/147), compared with 1.13:1 (35/31) in the macrolide-sensitive group. Significant differences across different isolation years, regions, and patient age groups were observed using the χ2 test (*P* < 0.05). All resistant strains were isolated post-2010, whereas sensitive strains demonstrated continuous temporal distribution. The highest prevalence of resistant strains was observed in Central China (37.9%, *n* = 151), whereas sensitive strains were more common in Eastern China. Furthermore, resistant strains were significantly more prevalent among infants younger than 6 months (51.0%, *n* = 203), as detailed in Table S3.

### Association between virulence genotyping and macrolide resistance

**Figure 2.**
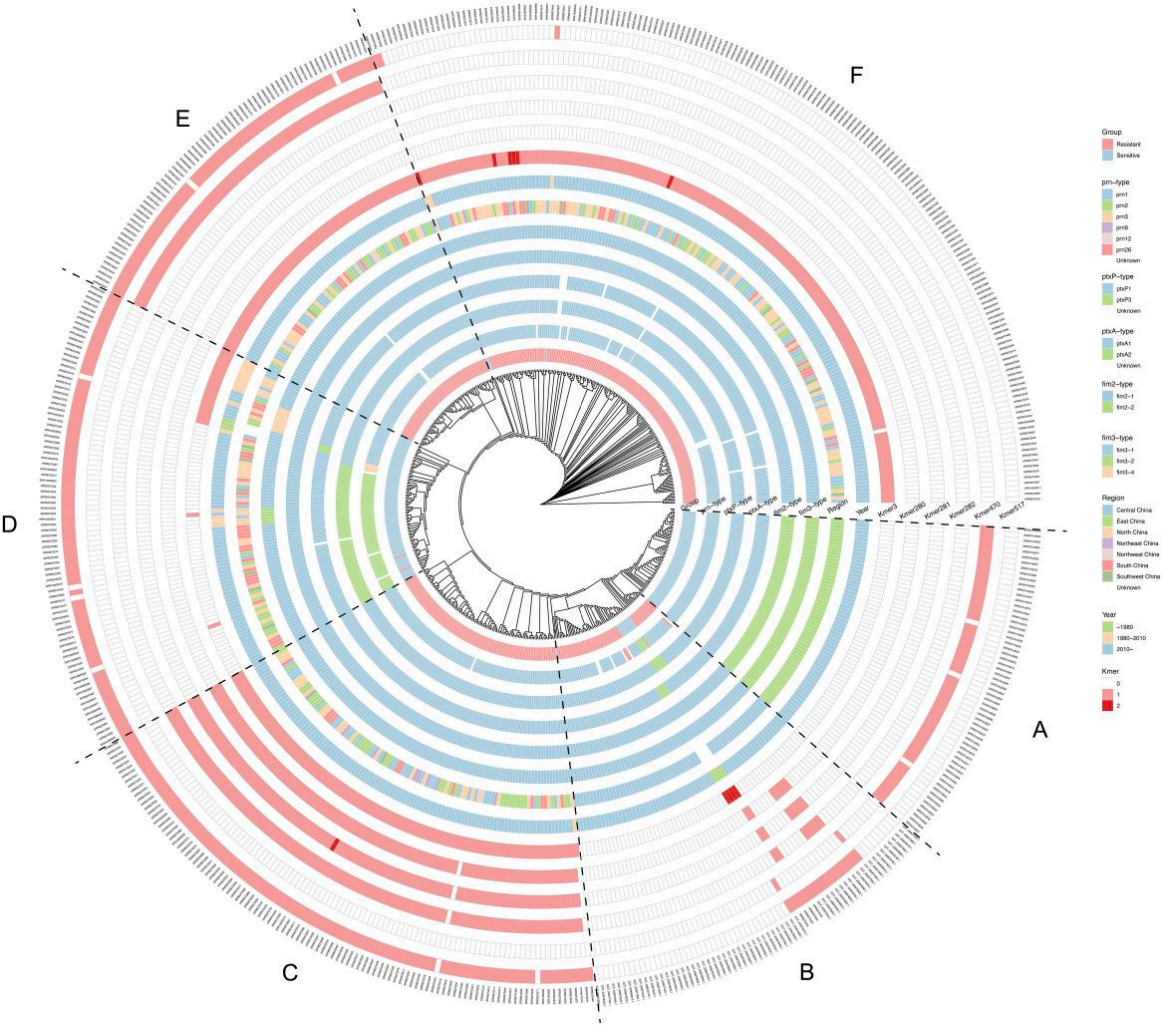
The phylogenetic tree is partitioned into six major clusters, labeled A through F, representing distinct evolutionary lineages of *B. pertussis* identified in this study. Phylogenetic tree based on whole-genome sequences, showing the genetic relationships among 556 *Bordetella pertussis* isolates linked to resistant-associated genetic variation. The radial-colored strips at the tree tips (inner to outer layers) correspond to: (i) virulence-associated subtypes of *Bordetella pertussis* isolates (*prn, ptxP, ptxA, fim2*, and *fim3*), (ii) epidemiological features (region and isolated year), and (iii) resistance-associated k-mers identified in the predictive model.

All strains were genotyped by analysing major virulence genes of *B. pertussis*, including *prn*, *ptxP*, *ptxA*, *fim2* and *fim3*. Among the macrolide-resistant strains, *prn1* (94.2%) and *ptxP1* (98.2%) were the predominant subtypes of *prn*-type and *ptxP*-type lineages. In contrast, *prn2* (39.2%) and *ptxP3* (41.8%) were found in the sensitive strains. *ptxA1* was dominant in both groups. For *fim2* and *fim3*, the subtypes *fim2-1* and *fim3-1* were common in both groups, but dominant in resistant isolates (100%) and less frequent in sensitive strains (53%–62%). Additional subtypes-including *ptxA2*, *fim2-2*, *fim3-2* and *fim3-4*, were found in the sensitive group, indicating a more diverse subtype distribution (Table S4). Notably, despite this overall diversity in virulence gene subtypes, the majority of sensitive isolates were phylogenetically clustered within two distinct branches, Clusters A and D (Figure 2), suggesting that these predominant sensitive lineages have diversified in their virulence gene profiles. Cluster A primarily exhibited *prn1*/*ptxp1*/*ptxA1*/*fim2-2*/*fim3-2*, with all isolates in this cluster originating from Shanghai and displaying close genetic relatedness.^15^ Cluster D was mainly characterized by *prn2*/*ptxp3*/*ptxA1*/*fim2-1*/*fim3-1*. The subtype combination *prn1*/*ptxP1*/*ptxA1*/*fim2-1*/*fim3-1* was the most common pattern in this study, accounting for 68.7% (382 of 556 isolates), and 96.6% (369 of 382 isolates) of these were macrolide-resistant (Table S5). Most resistant isolates were distributed across Clusters B, C, E and F, predominantly exhibiting the *prn1*/*ptxP1*/*ptxA1*/*fim2-1*/*fim3-1* pattern (90.0%, 369 of 410 isolates). The results indicated potential virulence gene subtype differences between macrolide-resistant and macrolide-sensitive strains.

### GWAS-based identification of differential k-mers associated with macrolide resistance

**Figure 3.**
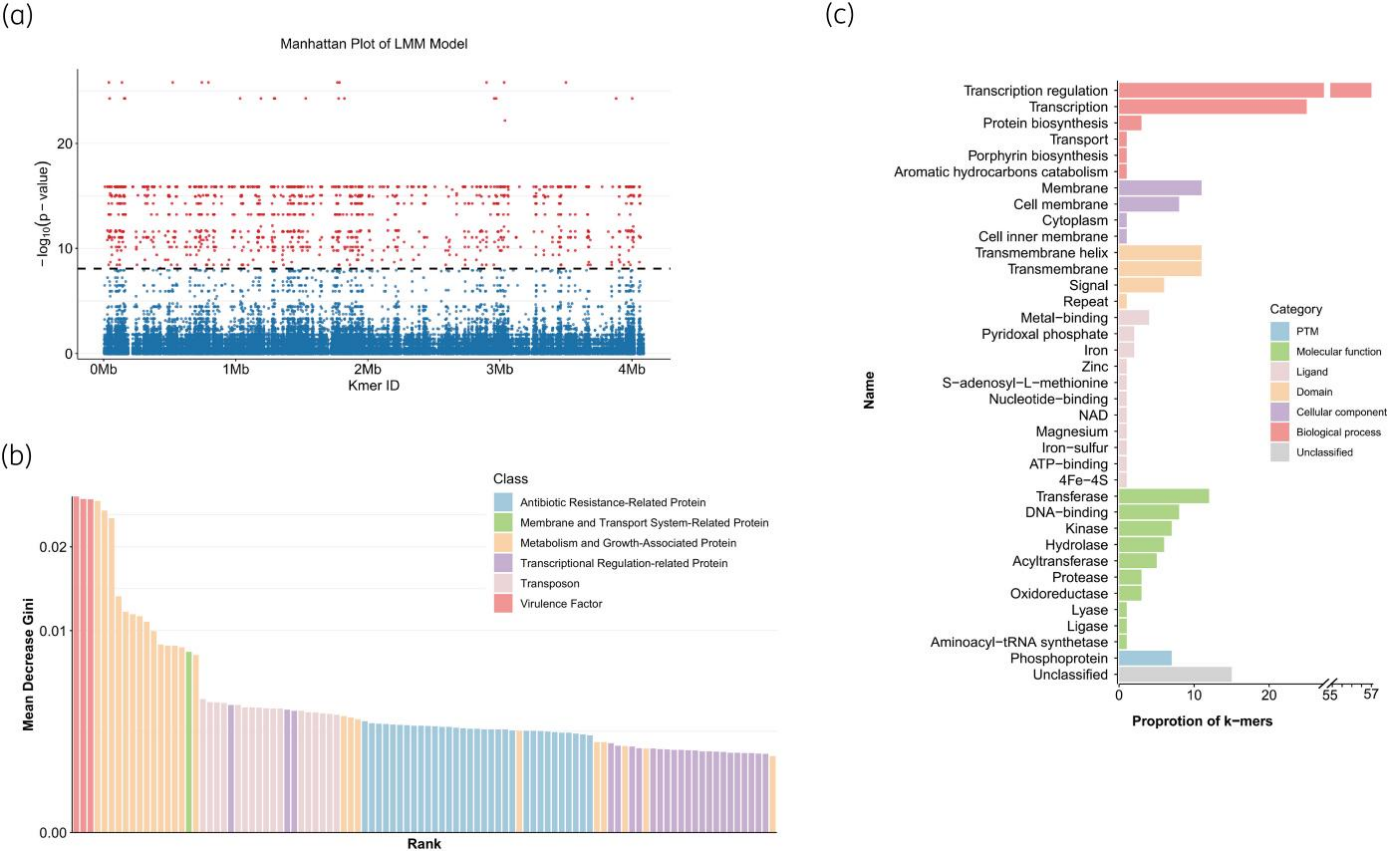
Resistance-associated k-mers identified by LMM model. (a) Genomic positions are plotted on the x-axis, with the dashed line denoting the significance threshold. Blue dots indicate all examined K-mer loci; red dots represent significant association loci with −log_10_(*P*) values above the threshold line. Manhattan plot of the LMM analysis showing k-mers with *P* < 8.34E-09 mapped to the reference genome GCF_004001123.1; (b) The top 100 k-mers sorted by MeanDecreaseGini (MDG); (c) Uniprot subsection of the top 100 k-mers predictors.

Assembly of 556 *B. pertussis* strains produced 10 099 268 k-mers, which were pre-filtered to 5 996 437 variants. Additional filtering using Pyseer's significance thresholds and removal of poorly fitting k-mers (‘bad-chisq’) yielded 1322 k-mers significantly associated with macrolide resistance (*P* < 8.34E-09). Among these, 1139 k-mers were mapped to the reference genome GCF_004008975.1 (Figure 3a).

**Figure 4.**
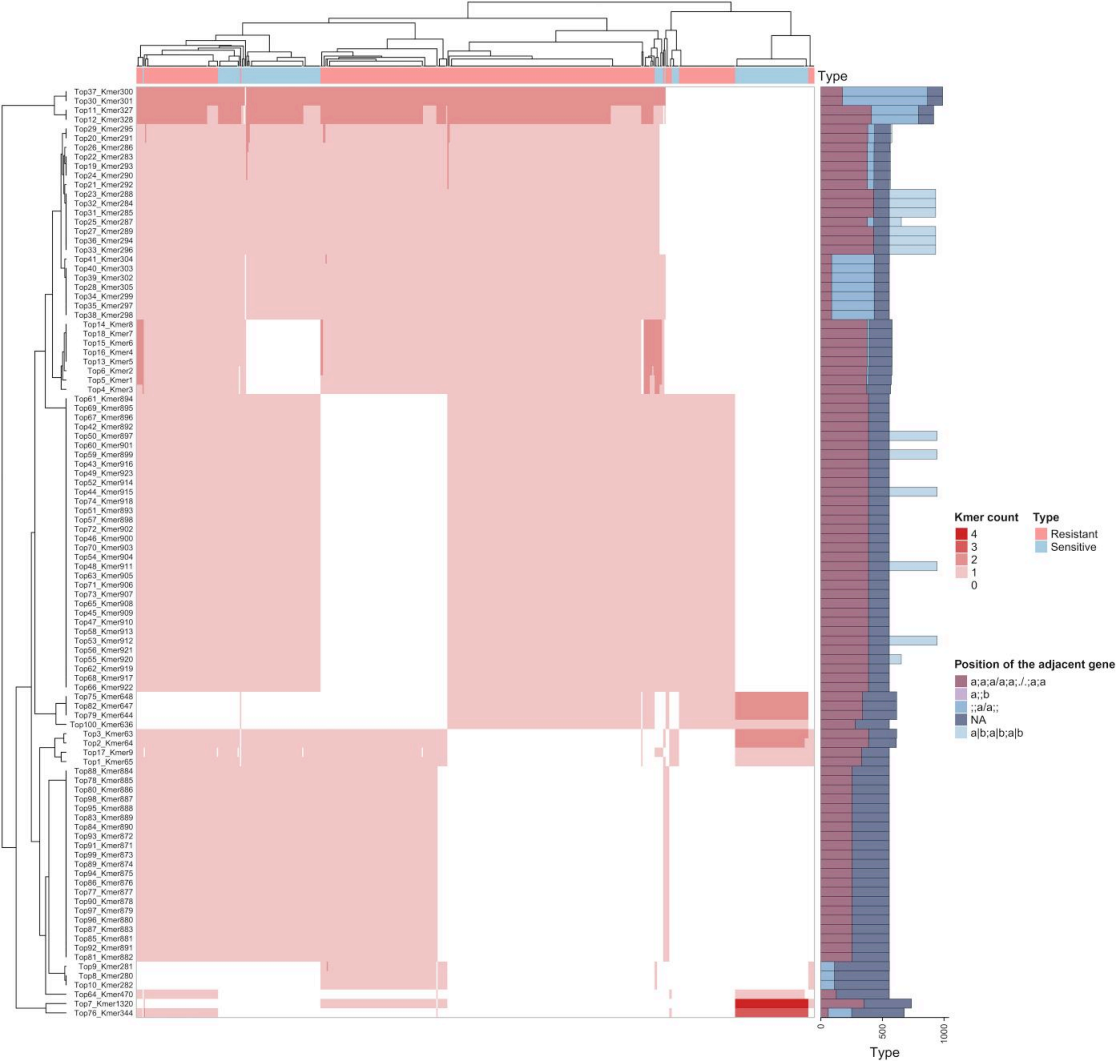
Heatmap of different k-mers relative to the position of the adjacent gene in different isolates. (a;a;a/a;a;./.;a;a) k-mer fully located within a gene’s coding region; (a; ;b) k-mer not aligned to any gene but flanked by genes immediately upstream and downstream; (;;a/a;;) k-mer unaligned to any gene and not near a gene on either side; (NA) k-mer is not located in any regions; and (a|b;a|b;a|b) k-mer is located within two different genes.

We annotated 1322 k-mers based on their position relative to the nearest gene as well as their frequency (Table S6). The k-mers clustering analysis showed that sensitive and resistant isolates formed distinct groups, which is congruent with the observed phylogenetic stratification. This suggests that k-mer patterns effectively capture lineage-specific genomic signatures associated with different resistance phenotypes. The presence of resistance-associated k-mers in intergenic regions highlights the potential involvement of regulatory elements in the genomic background of resistant isolates (Figure 4). Nevertheless, additional functional experiments are required to verify whether these non-coding variants directly drive antibiotic resistance.

Furthermore, the importance of all 1322 k-mers was evaluated using the VSURF algorithm, which assigned an MDG score to each. These k-mers were then ranked based on this score, followed by annotation and functional categorization (Table S7). Among the annotated proteins corresponding to the 1322-differential k-mers, the five most frequently represented proteins were BB0916 family autotransporter (7.8%, 103/1322), LysR-family transcriptional regulators (7.8%, 103/1322), CesT family type III secretion system chaperones (7.7%, 102/1322), formate dehydrogenase large subunits (7.6%, 101/1322) and membrane proteins (7.6%, 101/1322). Together, these categories accounted for 38.5% differential k-mers (Table S8).

After ranking all 1322 k-mers based on their MDG scores, the top 100 from this list were selected for further analysis due to their highest contributions to the model (Figure 3b). Based on the biological function of the proteins encoded by the genes containing these k-mers, the MarR family proteins, which are associated with antibiotic resistance, were mostly represented (32.0%, 32/100), followed by the LysR family (22.0%, 22/100) and *IS481* family transposases (17.0%, 17/100). Notably, the virulence protein BB0916 family autotransporter exhibited the highest contribution to the initial model; however, its function remains to be elucidated. Moreover, the top 100 k-mers were further categorized into six Uniprot subsections of functional predictors. Among these subsections, Biological Process was the most represented, with transcription regulation being the most frequent term (Table S9, Figure 3c).

### Model construction for macrolide resistance-associated isolates based on differential k-mer selection

**Figure 5.**
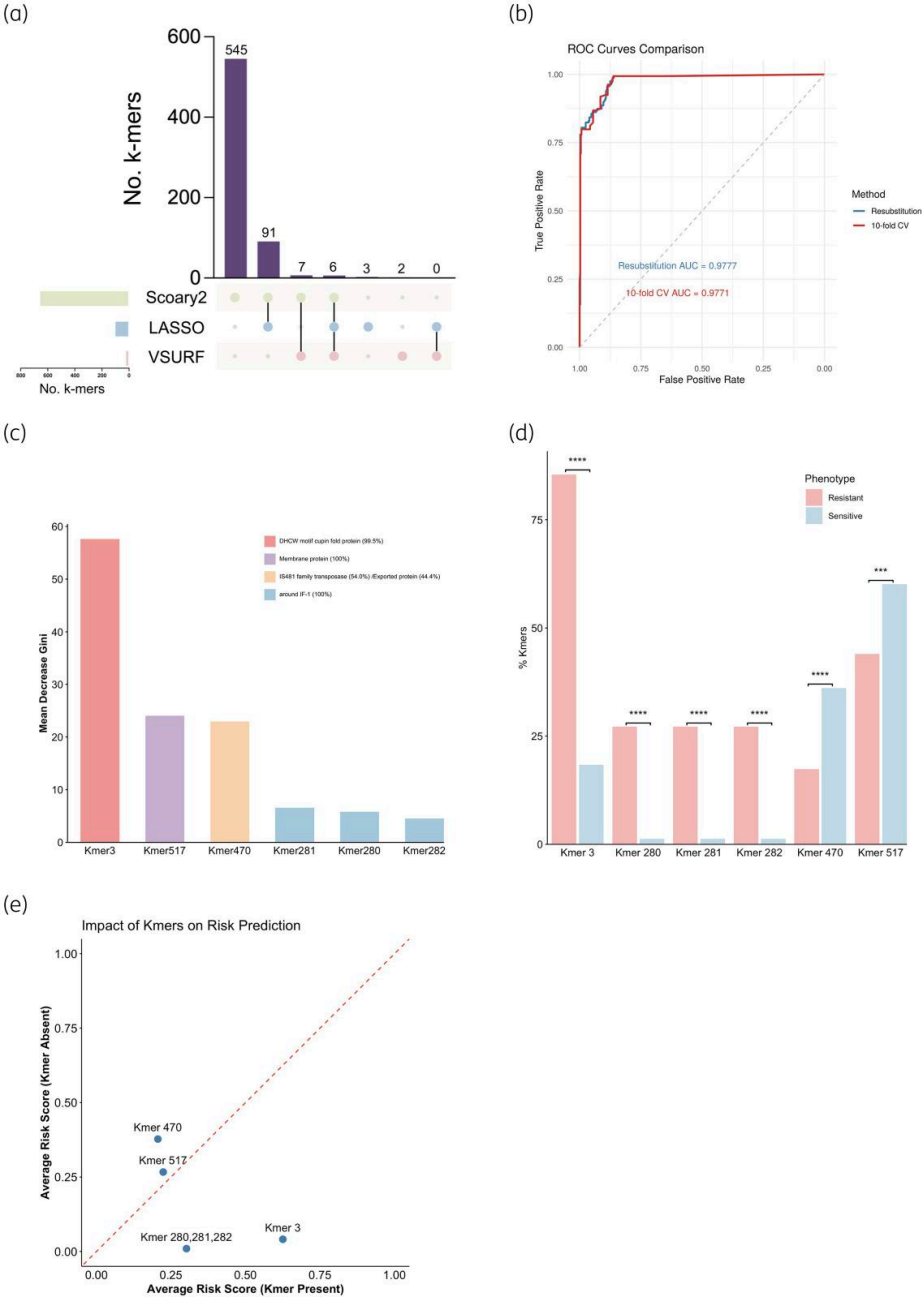
Model construction for macrolide-resistant strains based on differential k-mer selection by Scoary2, LASSO, and VSURF and predictive performance analysis of the simplified model. (a) The upset plot generated by each method; (b) Receiver operating characteristic (ROC) curve of the simple model (six k-mers); (c) Feature importance ranking based on the Random Forest MeanDecreaseGini (MDG). MDG measures the average reduction in node impurity contributed by a feature across all trees in the final simplified model (trained on the six-consensus k-mers). A higher MDG indicates greater importance in classifying resistant versus sensitive strains; (d) Comparison of the distribution proportions of the six k-mers between resistant and sensitive strains; (e) Regulatory effects of the presence or absence of the six k-mers on the average risk scores.

After identifying 1322 macrolide resistance-associated k-mers, we refined the key k-mers and minimized false positives by applying three distinct methods with their respective significance thresholds. Specifically, we identified 649 differential k-mers using Scoary2 (fisher_q < 0.05), selected 100 k-mers using LASSO (with both AUC_lambda.min and AUC_lambda.1se not equal to 0), and identified 15 differential k-mers using **VSURF** (as per its internal variable selection algorithm). The intersection of these independently filtered sets was then used to determine the final consensus k-mers (Table S10). To pinpoint the most reliable predictors, we determined the intersection of the outputs from these three complementary methods. This rigorous screening process resulted in six common k-mers (Figure 5a and b), which were then used to construct the simplified predictive model. The predictive performance of the simplified model, constructed using six k-mers, was compared with that of the initial model, which was based on 1322 k-mers. The simplified model demonstrated comparable sensitivity (97.7% versus 98.7%), specificity (92.1% versus 99.5%) and accuracy (93.4% versus 99.3%) compared to those of the initial model in the identification of macrolide-resistant strains (Table 1). The simple model comprising six k-mers, the classification performance for distinguishing between sensitive and resistant isolates was evaluated using a receiver operating characteristic (ROC) curve. The model achieved an area under the curve (AUC) of 0.98, showing excellent predictive accuracy (Figure 5c). The importance of each k-mer was ranked based on the mean decrease in Gini, revealing that k-mer_3 (DHCW motif cupin fold protein) contributed the most to the classification performance (Figure 5d, Table S11). Comparison of the presence rates of the six k-mers between resistant and sensitive groups showed significant differences, suggesting a strong association between these k-mers and resistance status. Importantly, k-mer_3 was present in 85.4% of resistant isolates but only 18.4% of sensitive isolates (*P* < 0.001) (Figure 5e, Table S12). The risk score analysis further quantified the contribution of each k-mer to resistance risk, demonstrating that the presence of k-mer_3 (DHCW motif cupin fold protein) was associated with an increased risk of resistance.

**Table 1.** Re-substitution estimate and cross-validation results based on random forest models

	1322 k-mers		6 k-mers	
	Re-substitution Estimate	10-fold CV Estimate	Re-substitution Estimate	10-fold CV Estimate
Accuracy	99.5	99.3	93.4	93.4
Balanced_Accuracy	99.3	99.1	94.9	94.9
Sensitivity	98.8	98.7	97.7	97.7
Specificity	99.8	99.5	92.1	92.1
PPV	99.4	98.7	78.6	78.6
NPV	99.5	99.5	99.3	99.3
Kappa^a^	0.99	0.98	0.83	0.83

NPV, negative predictive value; PPV, positive predictive value.

^a^Kappa values range from –1 to 1, with higher values indicating better agreement.

### Six K-mers in the simple model

Among the six k-mers ranked by importance, k-mer_3 exhibited the highest correlation (OR: 25.84, 95% CI: 15.58–44.03) (Table 2). Annotation of the k-mer_3 sequence revealed alignments to five distinct protein-coding genes in the pan-genome: DHCW motif cupin fold protein, Reductase, YedL, KdpC, and Fim2. Out of the 556 isolates identified, 553 (99.5%) were physically located within the coding region of the DHCW motif cupin fold protein gene. Further analysis revealed that 92.1% of the isolates harbouring this specific k-mer_3 exhibited a macrolide-resistant phenotype (340/369, *P* < 0.001), suggesting that k-mer_3 may be associated with macrolide resistance of *B. pertussis*.

**Table 2.** Association analysis between k-mers and macrolide resistance of *Bordetella pertussis* used in the simple model

	Macrolide-resistant (*n* = 398), *n* (%)	Macrolide-sensitive (*n* = 158), *n* (%)	*P* value	OR (95%CI)
kmer_3	340 (85.4)	29 (18.4)	<0.001	25.84 (15.58–44.03)
kmer_280	108 (27.1)	2 (1.3)	<0.001	28.96 (7.64–243.86)
kmer_281	108 (27.1)	2 (1.3)	<0.001	28.96 (7.64–243.86)
kmer_282	108 (27.1)	2 (1.3)	<0.001	28.96 (7.64–243.86)
kmer_470	69 (17.3)	57 (36.1)	<0.001	0.37 (0.24–0.58)
kmer_517	175 (44.0)	95 (60.1)	<0.001	0.52 (0.35–0.77)

**Figure 6.**
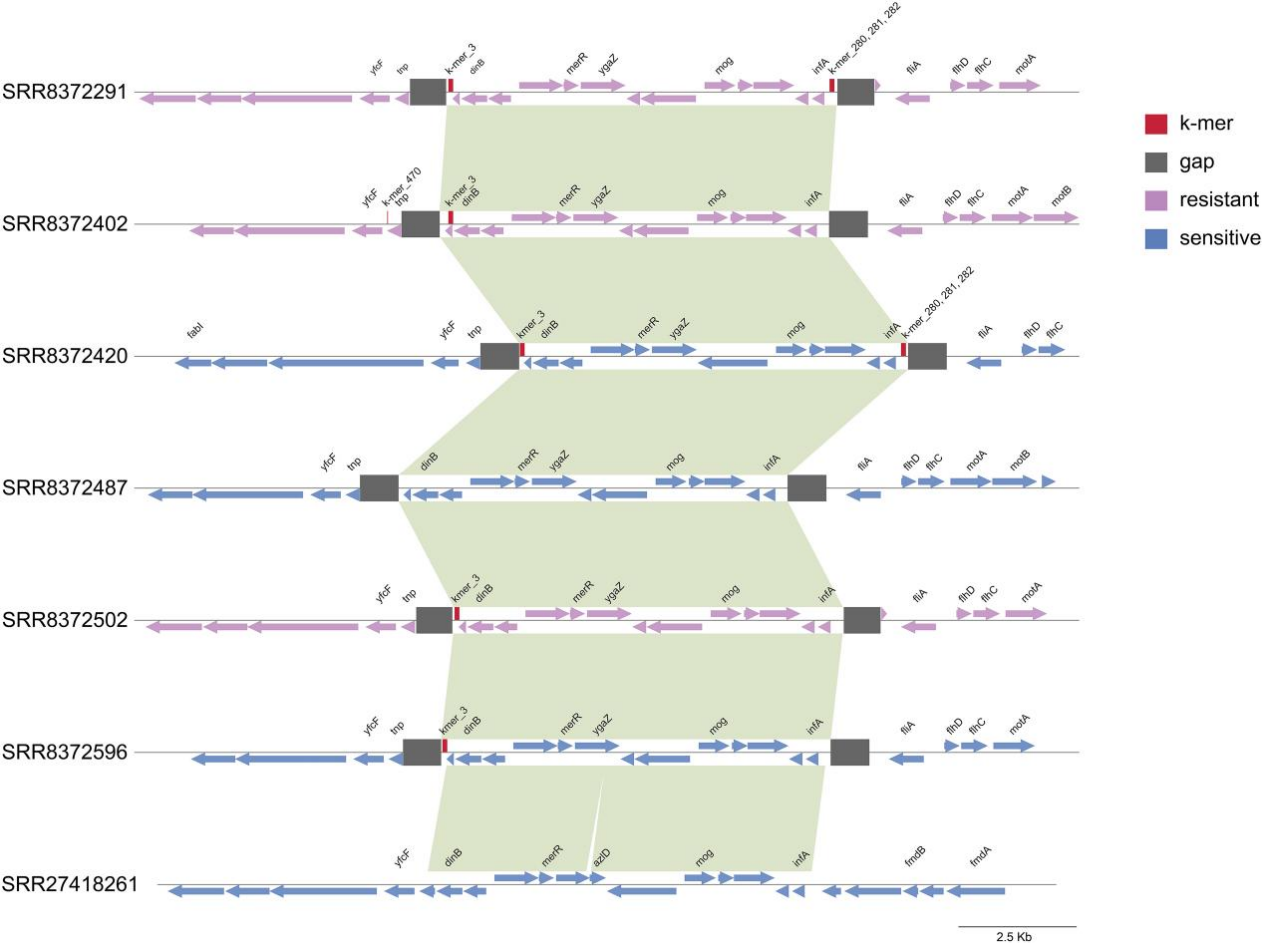
The insertion IS481 is located upstream of the *infA* gene and its associated genomic context. SRR8372291(cluster C, resistant); SRR8372402 (cluster E, resistant); SRR8372420 (cluster B, sensitive); SRR8372487 (cluster D, sensitive); SRR8372502 (cluster F, resistant); SRR8372596 (cluster D, sensitive); SRR27418261 (cluster A, sensitive).

Three characteristic k-mers (k-mer_280, k-mer_281, and k-mer_282) of the insertion sequence *IS481* were identified at the upstream of the *infA* gene in the reference genome. Multiple sequence alignment confirmed that these k-mers are specifically located within the *IS481* element (Figure 6). Analysis of 556 isolates revealed that strains carrying *IS481* exhibited a significantly increased antibiotic resistance rate of 98.2% (108/110, *P* < 0.001). Isolates containing these three k-mers are predominantly distributed within cluster C of the phylogenetic tree. Genome mapping of representative isolates selected through alignment showed that the *IS481* element displays a distinct genomic localization: its upstream end is adjacent to the *infA* gene, followed downstream by a conserved gene cluster. And it's downstream, followed by encoding flagellar-associated proteins. Although the genomic context flanking the *IS481* insertion site was conservative, the presence of this element is strongly associated with the antibiotic resistance phenotype of the strains (OR: 28.96, 95% CI: 7.64–243.86).

In addition, the genomic location corresponding to kmer_470 is not unique (54.0% *IS481* family transposase and 44.4% exported protein), and kmer_517 corresponds to the Membrane protein (100%). However, the contributions of both to the model are much lower than those of the above-mentioned k-mers.

### Other molecular characteristics of macrolide resistance in B. pertussis

The A2047G mutation in *B. pertussis* has been established as a critical determinant of macrolide resistance.^19^ A clear Association between this mutation and antimicrobial resistance phenotype was observed: all isolates harbouring the A2047G mutation exhibited resistance to macrolides (*n* = 280), whereas all isolates lacking the mutation were sensitive (*n* = 80). Notably, 195 isolates (3.0% of the cohort) with undetermined mutation status due to sequencing limitations were excluded from the final correlation analysis.

## Discussion

With the widespread use of macrolide antibiotics in treating *B. pertussis* infections, the issue of antimicrobial resistance has become increasingly prominent. The prevalence of macrolide-resistant strains varies among different populations, with distinct virulence characteristics observed among these strains.^20–23^ In this study, macrolide resistance was more frequently observed in infants younger than six months compared to older infants. Furthermore, the dominant virulence subtype pattern differed between resistance and sensitive strains (Figure 2, Tables S4 and S5). As treatment strategies must be adapted to specific resistance profiles, the rapid identification of macrolide-resistant *B. pertussis* strains is essential for effective clinical management.^24^ The primary known mechanism of resistance involves mutations in the 23S rRNA gene, which hinder the binding of macrolides to the bacterial 50S ribosomal subunit.^25^ Notably, increasing evidence shows that some resistant strains do not have the A2047G mutation, indicating that additional mechanisms may contribute to macrolide resistance.^11,12^ Other potential contributors, such as efflux pumps, acquired resistance genes, and regulatory elements affecting gene expression, remain insufficiently understood.^26^ The diversity of 23S rRNA gene copies may cause false-negative or false-positive results, limiting the reliability of mutation-based detection. While 23S rRNA mutation analysis is rapid and specific, it should be complemented by methods targeting other resistance mechanisms. Identifying genome-wide resistance markers and developing streamlined detection strategies are crucial for both mechanistic understanding and clinical application.

In this study, we analysed the whole-genome sequencing data of 556 *B. pertussis* isolates with confirmed macrolide resistance phenotypes, retrieved from the NCBI database. A k-mer-based genome-wide association study (GWAS) was performed using Pyseer, identifying 1322 k-mers significantly associated with resistance. To reduce false positives and enhance model efficiency, the results were further refined using three independent methods—Scoary2, LASSO, and VSURF—and six k-mers highly associated with the resistant phenotype were ultimately selected to construct a simplified prediction model. This simplified model demonstrated excellent classification performance (AUC = 0.98), comparable to the 1322-k-mer initial model in terms of sensitivity (97.7% versus 98.7%), specificity (92.1% versus 99.5%), and accuracy (93.4% versus 99.3%) (Table 1), highlighting its practical applicability.

Functional annotation of key k-mers within the initial model revealed several candidate features potentially linked to antimicrobial resistance. BB0916 family autotransporter proteins and CesT family type III secretion system chaperones, which are associated with virulence, were more prevalent in resistant isolates. Autotransporter proteins represent a secretion pathway in bacteria and may play important roles in host-pathogen interactions and virulence. However, to date, no studies have reported on the BB0916 family autotransporter proteins of *B. pertussis*.^27^ This suggests a possible interplay between virulence and resistance. The LysR-type transcriptional regulator system and membrane proteins were also frequently represented among the differential k-mers. Notably, the LysR system consistently appeared across multiple filtering strategies, suggesting it may play a conserved role in *B. pertussis* resistance pathways. LysR-type transcriptional regulators are global transcriptional factors known to be involved in various physiological processes and antibiotic resistance mechanisms in many pathogenic bacteria.^28^ They may influence bacterial virulence by modulating outer membrane protein expression or enhance antibiotic resistance by regulating the expression of resistance-related genes.^29,30^ However, their roles in *B. pertussis* remain largely unexplored, highlighting the need for further functional studies to elucidate their contribution to resistance development and pathogenicity in this species.^31^

We also identified several novel genomic features potentially involved in resistance. k-mer_3 is likely to encode a DHCW motif cupin fold protein. Cupin fold proteins exhibit diverse functions, including involvement in the degradation of aromatic compounds and redox reactions.^32,33^ However, the specific biological role of the DHCW motif cupin fold protein (k-mer_3) has not yet been reported. Another finding is an *IS481* insertion sequence located near the *infA* gene (k-mer_280/k-mer_281/k-mer_282). Despite the conserved synteny of the surrounding genomic region (*infA*, mog, and dinB), the insertion was strongly associated with resistance phenotypes. *IS481* is widely distributed across *B. pertussis* genomes, and its site-specific insertions can exert distinct regulatory effects on bacterial phenotypes. For instance, insertion of *IS481* within the *prn* gene disrupts pertactin expression,^34^ and insertion in the 5'-untranslated region (5'-UTR) of *bteA* modulates the expression of the type III effector protein BteA.^35^ Moreover, an insertion-driven antisense RNA has been shown to attenuate the expression of serotype 2 fimbriae and reduce the cytotoxicity of *B. pertussis*. This regulatory mechanism may represent a bacterial strategy for host adaptation and immune evasion.^36^ In light of our findings, the potential role of an *IS481* insertion near the *infA* locus in conferring macrolide resistance in *B. pertussis* merits further study.

Despite these promising results, our study has several limitations. First, the dataset lacks full epidemiological representation across geographic regions, time periods, and demographic groups, which may limit the generalizability of the model. Second, reliance on second-generation sequencing introduces challenges in resolving repetitive or structurally complex regions—areas where we identified possible non-coding k-mers associated with resistance. Third, differences in genome assembly methods—some downloaded from NCBI and others assembled *de novo* in this study—may have influenced the outcomes of the k-mer-based GWAS, especially given the evident clustering observed in the phylogenetic tree. Fourth, it should be noted that the above sequence-based limitations only serve as preliminary indications of genotype-phenotype associations; their findings still require further experimental validation and cannot replace necessary functional evidence.

### Conclusions

In summary, this study established a simplified and efficient k-mer-based model for identifying macrolide-resistant *B. pertussis* and uncovered several candidate resistance-associated loci. The model demonstrated excellent classification performance and provided important insights into potential resistance mechanisms. These findings may contribute to improved molecular diagnostics and inform the development of targeted treatment strategies.

## Supplementary Material

dkag131_Supplementary_Data

## Data Availability

The simulation experiment data used to support the findings of this study are available from the corresponding author upon request.

## Supplementary data

Tables S1–S12 are available as Supplementary data at *JAC* Online.
